# Developing and evaluating per-protocol adherence definitions in a large pragmatic clinical trial: insights from the Diuretic Comparison Project

**DOI:** 10.1515/med-2026-1514

**Published:** 2026-10-12

**Authors:** Sonia T. Anand, Cynthia Hau, Michael J. Davenport, Areef Ishani, William C. Cushman, Peter A. Glassman, Addison A. Taylor, Robert A. Lew, Ryan E. Ferguson, Sarah M. Leatherman

**Affiliations:** Cooperative Studies Program Coordinating Center, VA Boston Healthcare System, Boston, MA, USA; Minneapolis VA Healthcare System, Minneapolis, MN, USA; Department of Medicine, University of Minnesota, Minneapolis, USA; Medical Service, Memphis VA Medical Center, Memphis, TN, USA; Department of Preventive Medicine, University of Tennessee Health Science Center, Memphis, TN, USA; Department of Veterans Affairs, Pharmacy Benefits Management Services, Washington, DC, USA; VA Greater Los Angeles Healthcare System, Los Angeles, CA, USA; David Geffen School of Medicine at UCLA, Los Angeles, CA, USA; Michael E. DeBakey VA Medical Center, Houston, TX, USA; Department of Medicine, Baylor College of Medicine, Houston, TX, USA; Department of Biostatistics, Boston University School of Public Health, Boston, MA, USA; Department of Medicine, Boston University Chobanian & Avedisian School of Medicine, Boston, MA, USA

**Keywords:** adherence, per-protocol, nonadherence, real-world data, pragmatic trial, electronic health records

## Abstract

**Objectives:**

Pragmatic clinical trials leveraging real-world data, such as the Diuretic Comparison Project (DCP), present unique challenges in measuring treatment adherence compared to traditional randomized controlled trials. In DCP, medications were managed through routine VA care and monitored via pharmacy claims, requiring tailored methods to assess adherence beyond standard measures like medication possession ratio (MPR). To develop a data-driven approach to define protocol treatment adherence for pragmatic comparative effectiveness trials.

**Methods:**

Structured EHR pharmacy dispense records guided categorization of treatment changes for two diuretics – chlorthalidone (CTD) and hydrochlorothiazide (HCTZ). We examined fill intervals, total drug supply, filling patterns among others to identify prescribing characteristics informing medication adherence definitions and time censoring for per-protocol analysis.

**Results:**

Nonadherence was defined as having a gap (>90-days pause in assigned treatment), switch (receiving the other study medication), or discontinuation (without CTD/HCTZ for >90-days). 5,476 patients met nonadherence criteria – 2,851 (52 %) in CTD and 2,625 (48 %) in HCTZ. Switching occurred earlier in CTD patients, gaps were more common in HCTZ, but discontinuation was similar between groups.

**Conclusions:**

Defining adherence requires complex decision-making hybrid framework entailing both clinical and statistical expertise. Pragmatic studies should allow flexibility to generate data-driven, clinically-sensitive measures that appropriately describe trial compliance.

## Introduction

Per-protocol analysis is a clinical trial procedure to compare treatment differences in patients who do and do not adhere to the study interventions as outlined in the study protocol. In comparative effectiveness research the classification of treatment compliance is an important study component because it shows a reflection of the effectiveness of therapies in the real-world setting. Traditional randomized controlled trials (RCT) often incorporate standardized testing or questionnaires (such as pill counts and patient diaries) to generate generally accepted adherence metrics that are typically sufficient to support per-protocol analyses. However, ascertainment of treatment adherence may not be as straightforward in pragmatic trial settings with clinically embedded study interventions and real-world data to detect changes in treatment adherence.

The Diuretic Comparison Project (DCP), a Veterans Affairs (VA) Point-of-Care pragmatic clinical trial, was conducted to compare relevant clinical outcomes between hydrochlorothiazide (HCTZ) or chlorthalidone (CTD) for hypertension treatment [1], 2]. Both drugs are approved and long-used in clinical practice as first-line antihypertensive treatment. However, a significantly higher proportion of patients have historically received HCTZ [3], despite some studies suggesting CTD might be the preferred agent [4], 5]. DCP was designed to address this pivotal clinical question.

DCP was performed with study procedures embedded within the VA Healthcare System. Over thirteen thousand older hypertensive patients were randomized across the US to HCTZ or CTD [2], 6]. Trial data was collected exclusively from electronic health records (EHR), National Death Index (NDI) and administrative claims generated in real-world clinical settings. The primary outcome trial analysis was based on intention-to-treat, but a per-protocol analysis was prespecified to compare the effectiveness of CTD to HCTZ in preventing major adverse cardiovascular events and non-cancer deaths.

The DCP protocol defined treatment adherence as a medication possession ratio (MPR) of ≥80 %, a common threshold used for antihypertensive treatment. However, MPR (calculated as total numbers of days of drug supplied divided by days in study) is susceptible to misclassification as it doesn’t consider gaps in medication fills or switching of medication, jeopardizing the accuracy of per-protocol effect estimates. As such, further work was needed to refine the definition of medication adherence when using real-world pharmacy data.

We accordingly conducted a per-protocol analysis, using the trial protocol to clearly define what qualifies as treatment adherence, as well as what constitutes deviations that could affect treatment effectiveness [7]. For the per-protocol analysis, we used inverse probability weighting to adjust for the prognostic and time-varying variables predicting adherence to the protocol and has been described in further detail in our primary DCP per-protocol paper [7]. The g-methods used in the per-protocol analysis further addresses biases related to informative censoring and time-dependent confounding. We found that the estimated five-year risk ratio was 1.36 (95 % CI 0.96–2.12) for those taking chlorthalidone for the composite primary outcome of nonfatal cardiovascular disease and noncancer related death compared with hydrochlorothiazide [7].

This methods manuscript focuses on describing the strategy for ascertaining study medication adherence from outpatient pharmacy records collected using DCP as an example. Our report delineates the learning and decision-making process, as well as presenting our methods in applying a data-driven approach to define adherence to treatment with real-world pharmacy data for embedded pragmatic clinical trials.

## Methods

### Diuretic Comparison Project (DCP)

DCP randomized 13,523 older hypertensive patients from 72 participating VA medical centers. Participants were randomly assigned to continue with their existing HCTZ (25 or 50 mg) or switch to a dose equivalent CTD (12.5 or 25 mg, respectively). The primary outcome was the first occurrence of a composite cardiovascular outcome consisting of nonfatal myocardial infarction, stroke, hospitalization for heart failure, urgent coronary revascularization for unstable angina, and non-cancer death.

The initial fill of randomized medications was initiated by study staff and filled through normal pharmacy mechanisms. After randomization, prescriptions of CTD and HCTZ were managed by primary care clinicians who could make necessary modifications to the allocated treatment as needed according to usual care. Randomization to the different study drugs were to equipotent doses. Patients were responsible for initiating the fill of subsequent prescriptions through outpatient pharmacy services. Prescription data, including those used to define adherence (e.g., prescribed drug name, date, dose, and coverage) were documented in the EHR by healthcare providers. Data used in the study was then extracted from EHR as contained in the VA Corporate Data Warehouse (CDW). Prescriptions issued in an inpatient setting were excluded from the evaluation of adherence to study interventions intended for dispensing at outpatient pharmacies. DCP was approved by the VA Central Institutional Review Board (CIRB), with waivers of consent documentation and Health Insurance Portability and Accountability Act (HIPAA) authorization. The trial was registered at ClinicalTrials.gov (NCT02185417).

### Measuring adherence

A combination of tools was used to define treatment adherence, including definitions in the protocol, discussions with clinical experts (consisting of cardiologists, nephrologists, epidemiologists/biostatisticians) about what would be classified as adherent in clinical care, and fill patterns present in the pharmacy data.

As treatment beyond randomization was in the purview of a randomized participant’s primary care provider, detailed discussions were conducted with the trial investigators and steering committee regarding strategies for ascertaining medication adherence in clinical care. As adherence to the therapy can be based on factors such as laboratory results, patient preference, prescriber preference, adverse events, allergic reactions, etc., these discussions included such considerations in determining the criteria for defining treatment adherence.

Descriptive summary statistics, such as percentages and counts, were performed on pharmacy data collected during the study period to characterize common durations between fills, fill patterns for confirmed treatment switches and discontinuations.

Upon combining clinical discussions with descriptive data summaries, classification strategies were developed to incorporate scenarios that would constitute medication adherence, which included (1) maximum time allowance between refills (gaps), (2) reasonable frequencies of switching between study medications (switches), and (3) defining discontinuation of both CTD and HCTZ based on time elapsed since last fill (discontinuation).

### Study outcomes

The intervals between study randomization and end of trial follow-up were determined for each participant, in addition to defining the durations where patients were still adhering to the randomized treatment intervention. Participants were considered as reaching the end of trial follow-up when a primary outcome (composite of nonfatal MI, stroke, HF resulting in hospitalization, urgent coronary revascularization for unstable angina, and noncancer-related death), withdrawal, or death was detected (whichever occurred first); otherwise, the end of trial follow-up would default to the study completion date (06/01/2022). During the per-protocol analysis, patients were censored if the participants deviated from their randomized treatments based on the nonadherence definitions of gaps, switches and discontinuation.

Prior to censoring, we assessed the potential loss of outcomes depending on when a patient was considered adherent. We looked at the number of outcomes based on whether we assessed gaps using a 30-day, 60-day, and 90-day gap.

### Analysis

We performed descriptive summary analyses to assess the counts and frequencies of outcomes that occur before and after gaps, switches, and discontinuation. We also evaluated summary measures of gaps, switches, and discontinuation and overall adherence among those who met the criteria for adherence.

In addition, we conducted an unadjusted nonadherence analysis assessing the Cox Proportional Hazard Ratio of nonadherence among those taking CTD compared to those taking HCTZ.

## Results

To define the different metrics for adherence in participants, we assessed the pharmacy data patterns combined with clinical input.

### Real-world prescription practice

HCTZ and CTD were usually prescribed for covering a 90-day supply. On some occasions, the structured pharmacy data showed extra supply (range 1–90 days) was dispensed to participants in advance of an expected refill based on previously dispensed quantity. Reasons for prescribing supplementary quantities were potentially documented in clinical notes, though such adjudication requires extensive chart review and record processing time that is beyond the scope and resourcing of this analysis. Officially documented discontinuation of medication was also rarely seen in the pharmacy records and requires proper defining from a research perspective.

### Gaps

We evaluated the average days of fills across the cohort to decide on a cut-off. Within the pharmacy fill data, 96 % of fills were for 90 days, 3 % were for 30 days, and 1 % for fills 1–89 days (not including 30 days). Implementing a 90-day cutoff allows us to capture about 85 % of individuals who go on to have subsequent prescription refills. Using a 30-day or 60-day cut-off, we capture only 50 and 70 %, respectively. A 90-day cut-off to define treatment discontinuation and gaps in adherence is consistent with both clinical input and previous studies analyzing diuretics for antihypertensive treatment [8], 9]. By the end of the study, we had a total of 13.2 % who had a gap in the CTD group and 16.9 % in the HCTZ group (Table 1 and Figure 1).

**Table 1:** Descriptive details of adherence metrics for each treatment by number of follow up years since randomization.

Treatment group	Number of follow up years	Total patients left after follow up period^a^	Patients with switch^b^			Patients with gap^b^			Patients with discontinuation^b^		
			n	Cumulative n	Cumulative % of total randomized	n	Cumulative n	Cumulative % of total randomized	n	Cumulative n	Cumulative % of total randomized
CTD	**Randomization**	**6,756**									

CTD	Less than 1 year	4,571	585	585	8.7 %	403	403	6.0 %	407	407	6.0 %
CTD	1–2 years	2,405	69	654	9.7 %	308	711	10.5 %	344	751	11.1 %
CTD	2–3 years	1,139	22	676	10.0 %	128	839	12.4 %	206	957	14.2 %
CTD	3–4 years	263	13	689	10.2 %	47	886	13.1 %	69	1,026	15.2 %
CTD	4–5 years	31	2	691	10.2 %	8	894	13.2 %	14	1,040	15.4 %

HCTZ	**Randomization**	**6,767**									

HCTZ	Less than 1 year	4,815	107	107	1.6 %	598	598	8.8 %	410	410	6.1 %
HCTZ	1–2 years	2,467	41	148	2.2 %	357	955	14.1 %	390	800	11.8 %
HCTZ	2–3 years	1,197	19	167	2.5 %	138	1,093	16.2 %	182	982	14.5 %
HCTZ	3–4 years	264	6	173	2.6 %	44	1,137	16.8 %	76	1,058	15.6 %
HCTZ	4–5 years	39	2	175	2.6 %	4	1,141	16.9 %	16	1,074	15.9 %

^a^Takes into account those who were censored due to switch, gap, discontinuation, study outcome, death due to non-study outcome, reaching study end date, no medication fill after randomization, no medication fill for the randomized medication, and withdrawals. ^b^Mutually exclusive groups. Randomization: N of patients randomized.

**Figure 1:**
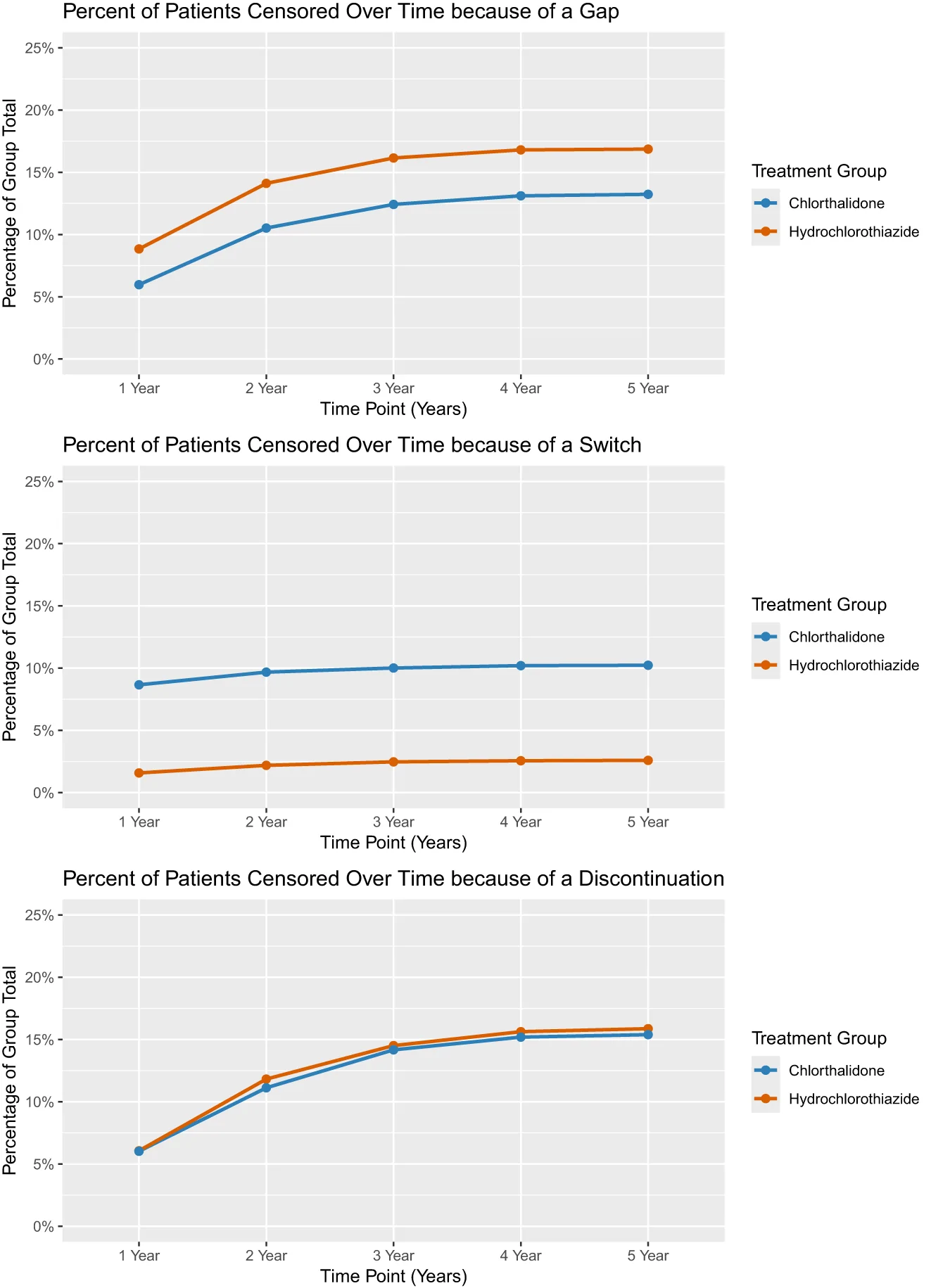
Percentage of patients censored due to gap, switch or discontinuation over time by treatment group.

We initially decided to use a 90-day gap window based on clinical input but conducted separate analyses to see how a 30/60-day gap would affect outcomes. Ultimately, we did not find compelling evidence that using 30/60-day windows were appropriate. The summary table can be seen in Table 2. Prior to flagging for nonadherence, we evaluated the number of primary outcomes before and after gaps, switches, and discontinuation. There were 72 outcomes before a gap and 151 outcomes after a gap.

**Table 2:** Frequency of primary outcomes and other reasons when using 30-day, 60-day, and 90-day for censoring of gaps.

	Primary outcome	Other outcome^a^	Study end^b^	Discontinuation	Switch
90 day gap	915	100	7,404	2,114	866
60 day gap	825	96	6,368	1,899	818
30 day gap	667	72	3,864	1,468	714

^a^Other outcome=non-study related deaths. ^b^End of the study.

### Switches

We assessed the number of switches patients were having, followed by the duration of fills between switches. The decision to use 30 days was a clinical decision as an average refill for medication is 30, 60 or 90 days and thus anyone changing between medications for less than 30 days is not truly intending to switch medications. If the switch occurred for a duration of less than 30 days before returning to the original medication, then it was not considered a switch. This was determined through validation/chart review of switch cases where there was switching back and forth. Based on the time between the switching, it was decided that 30 days was consistent with an actual switch. Within the first two years since randomization, about 10 % of patients in the CTD group switched to HCTZ and 2 % in the HCTZ group switched to CTD. Over the course of the trial of 5.3 years, there were a total of 691 patients who switched from CTD (randomized drug) to HCTZ and 175 patients who switched from HCTZ (randomized drug) to CTD (Table 1 and Figure 1). For outcomes in terms of switching, we had 29 outcomes before a switch and 85 outcomes after a switch. The average number of days an outcome happened before a switch was 255 (range 7–708 days) and the average number of days an outcome happened after a switch was 469 (range 2–1,410 days).

### Discontinuation

For defining discontinuation, we assessed the time from the last study drug filled and the study end time for the patient. If there were no studies drugs dispensed for >90 days prior to the end of the study, then the patient was considered discontinued. The counts included those changed to another antihypertensive drug class. In the first two years, about 11 % of participants in the CTD group and 12 % in the HCTZ group discontinued the study medications. By the end of 5 years, about 16 % of participants in both CTD and HCTZ group had discontinued (Table 1 and Figure 1). When assessing outcomes, there were 376 primary outcomes before discontinuation and 280 outcomes after discontinuation.

### Overall adherence

We defined per-protocol adherence for this pragmatic trial using a static treatment strategy. Due to the lack of data on reasons for gaps, switches, or discontinuations, participants were deemed nonadherent if they experienced a gap, switch or discontinuation, regardless of the reason. Adherence was defined as continuous use of the randomized treatment without any gaps, switches, or discontinuation.

There was a total of 6,756 participants randomized to chlorthalidone and 6,767 randomized to hydrochlorothiazide in DCP. Overall, 8,047 (60 %) patients met the definition of per-protocol adherence with 3,905 (57.8 %) in the CTD group and 4,142 (61.2 %) in the HCTZ group. Those who never received a fill for the medication they were randomized to (n=32) and those who never had a study medication fill after being randomized (n=56) were censored at the start of the study and excluded from these analyses. Overall, there were about 6 % of participants who had a switch, 15 % with a gap, and 16 % with discontinuation. When censoring participants at the first instance of nonadherence, 462 patients were censored before they experienced a primary outcome and 372 were censored after having a primary outcome.

Additionally, those taking CTD had a 15 % increased risk of nonadherence compared to those taking HCTZ (HR: 1.15; 95 % CI 1.09–1.21) (Appendix Figure A). Patients were 15 % more likely to make changes to their randomized medication if randomized to chlorthalidone.

## Discussion

Overall, for our pragmatic trial, we established a rigorous and pragmatic definition of treatment adherence by integrating pharmacy fill data with clinical input, focusing on medication gaps, switches, and discontinuations. Pharmacy data revealed that nearly all fills were for 90 days, so we used a 90-day gap in refills as our primary cut-off for defining interruptions in medication use; this approach aligns with clinical practice and existing literature. We also evaluated alternative thresholds (30- and 60-day gaps), but these captured fewer adherence lapses and were not supported by clinical consensus. Using the 90-day window, gaps in adherence occurred in 13.2 % of the chlorthalidone group and 16.9 % of the hydrochlorothiazide group. Switches between study medications were classified as a true switch only if the new medication was taken for more than 30 days, based on typical refill cycles and clinical reasoning. Switches occurred in about 10 % of CTD users and 2 % of HCTZ users switching within two years. Discontinuation was defined as no further dispensing of study medication for more than 90 days prior to the study’s conclusion. Discontinuation rates were similar between groups, affecting about 16 % of participants over five years. Using this structured approach – focusing on 90-day gaps, ≥30-day switches, and 90-day discontinuations – we then operationalized per-protocol adherence as uninterrupted use of the randomized medication without qualifying gaps, switches, or discontinuation. This framework allowed us to systematically assess and compare adherence rates between study groups and minimize misclassification due to differences in refill patterns or temporary changes.

Our pragmatic clinical trial captured the full spectrum of real-world data characteristics, encompassing associated limitations, adaptable study design, and substantial data volume. We presented our methods of using a data-driven approach to define medication adherence. Decisions made for defining adherence relied on a combination of the protocol, clinical insight, and review of the trial’s pharmacy data. Definitions of medication gaps, switches and discontinuation were both empirically derived and based on clinical knowledge of prescribing practices. Adherence was defined using gaps, switches, and discontinuation and this definition of adherence was used in the per-protocol analysis of the DCP trial. The final adherence criteria were developed with considerations of EHR data availability and validity, as well as clinically meaningful targets for oral antihypertensive regimens and the DCP trial design and protocol specifications.

We needed to ensure the guidelines we were setting forth to identify medication fill gaps, switches and discontinuations were consistent with pharmacy data and usual clinical practice. We first considered the number of patients that would be excluded from analysis for fill gaps of 30-, 60-, and 90-days. Most medication fills for CTD and HCTZ were for a 90-day supply of medication. Using observed data and clinical input, a gap in medication was defined as the first instance of having more than 90 days between refills of randomized study medication. A medication switch was defined as the initial point at which a patient transitioned from their randomized study medication to the alternative study medication and the patient did not switch back to their randomized treatment for more than 30 days based on clinical decision of how usual refills are given for 30-, 60, or 90-days and changes under 30 days indicate an unintentional switch. Treatment discontinuation was defined as a lack of medication fill of either study medication for more than 90 days prior to study end based on clinical input and other diuretic studies [8], 9].

Due to the half-life of the medications, we defined time to event in our per-protocol analysis based on when the first gap or switch between medications occurred for a patient [7]. Allowing for multiple gaps or multiple switches would make it difficult to determine a causal relationship between the primary outcome and study treatment.

Even though there are some details outlined specific to the DCP trial, it should be noted that the process of developing the definition for adherence to further carry out a per-protocol analysis in a pragmatic trial remains the same: in-depth conversations with clinical experts on the trial protocol criteria, assessment of the available EHR data, use of proxy variables when specific variables are not available in the data, using a combination of clinical input and data to drive the decision-making process, and finally performing any sensitivity analyses that allows for analyzing the data with different cut-offs or criteria that are relevant to the study question. Some of our processes may be specific to the VA system such as prescription refill practices, compliances of clinic visits, etc. and as such should be assessed carefully around the available data and populations being studied to develop adherence metrics. Defining adherence should also be built into the protocol along with accounting for switches, gaps, and discontinuation when planning sample size. Protocol development should account for drug discontinuation that occurs during inpatient hospitalization, incorporating real-time monitoring for these events while preserving the pragmatic design of the trial. Additionally, the use of more sophisticated methodologies to refine the definition of adherence in pragmatic trials based on the study protocols can be used when unstructured data such as EMR notes are available for analysis. These methods can include natural language processing (NLP) and large language models (LLMs) that allow for finding specific reasons for adherence to treatment strategies like why they stopped a medication or switched medications. For our specific study, the use of these tools and the resources was not available but will be beneficial in future studies.

Our study had a few limitations. As a pragmatic trial, prescription behaviors after randomization were left up to the prescribing physician and the patient. Thus, it was difficult to ascertain reasons for medication changes without extensive chart review. Reliance on structured EHR data limits our ability to identify the underlying reasons for gaps, switches, or discontinuations in medication use, as well as the impact of potential hospitalizations and other events that could interrupt medication use. This could have also caused informative censoring bias as reasons for changes could have been due to health risks or medication intolerances but the use of inverse probability censoring weighting in the primary per-protocol analysis accounts for this bias. While the incidence of all-cause hospitalizations did not significantly differ between the treatment groups [6], the current adherence determinations did not account for medication interruptions occurred during hospital stays. Clinical visits were not protocolized leaving the decision to define time on medication to algorithmic methods. The lack of protocol-driven visits, however, allows for defining real-world adherence more objectively because the clinical actions and data are not impacted by the trial.

## Conclusions

In conclusion, the proposed framework provides a practical and clinically informed approach for operationalizing treatment adherence in pragmatic trials using structured pharmacy data; however, further validation and methodological refinement are warranted. Despite challenges such as non-protocolized visits and varying prescription behaviors, our approach provided meaningful insights into medication gaps, switches, and discontinuations, setting a strong foundation for future studies leveraging advanced methodologies.

## Figures and Tables

**Figure A:**
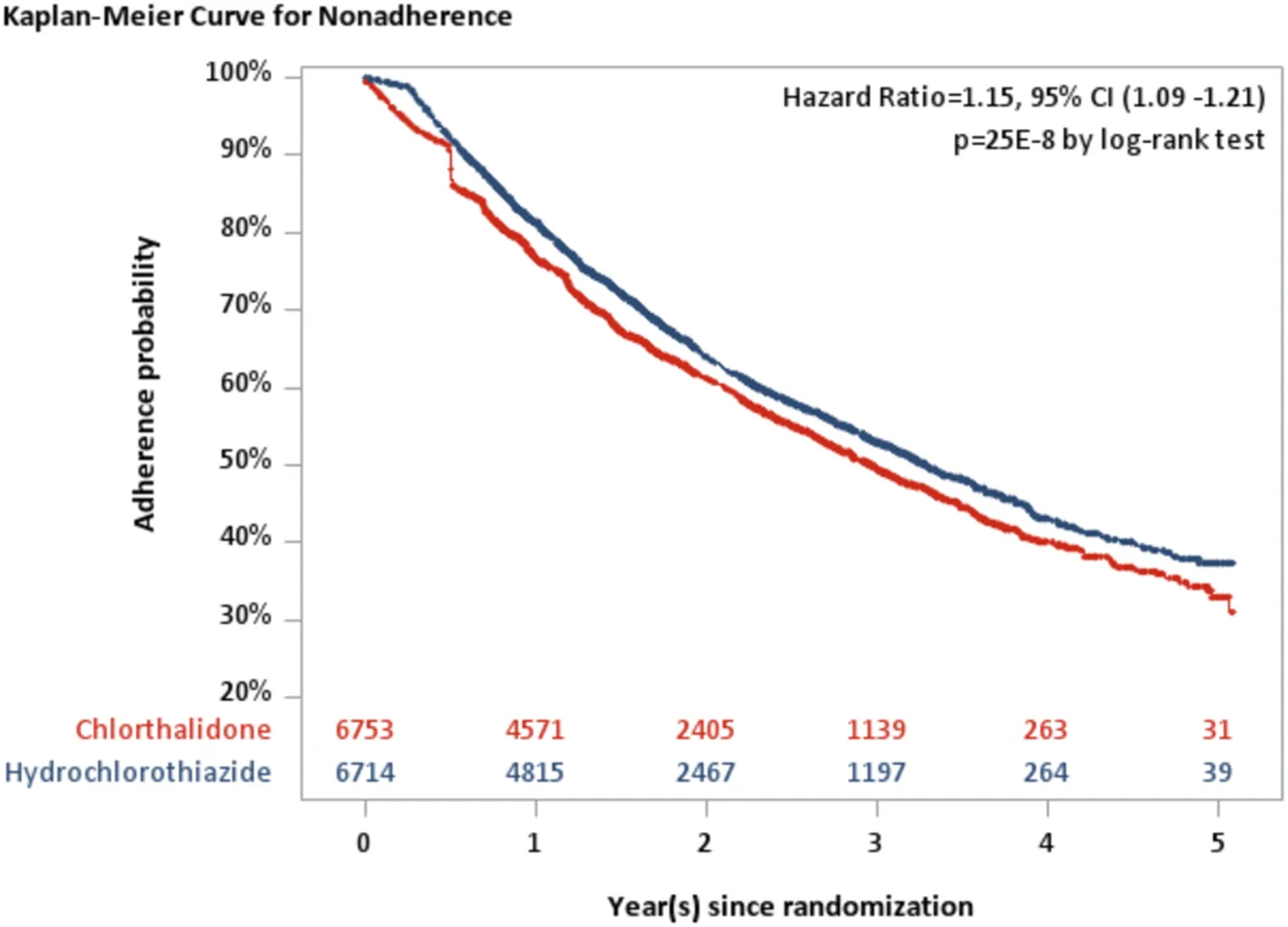
Kaplan-Meier curve for nonadherence by treatment group.
